# Beyond bed counts: equity and access to adult intensive care in middle-income countries, the Brazilian case

**DOI:** 10.62675/2965-2774.20260158-2

**Published:** 2026-08-21

**Authors:** Ederlon Rezende, Cristiano Augusto Franke, Patrícia Machado Veiga de Carvalho Mello, Jorge Ibrain Figueira Salluh, Rui Moreno

**Affiliations:** 1 Hospital do Servidor Público Estadual de São Paulo "Francisco Morato de Oliveira Intensive Care Department São Paulo SP Brazil Intensive Care Department, Hospital do Servidor Público Estadual de São Paulo "Francisco Morato de Oliveira - São Paulo (SP), Brazil.; 2 Hospital de Pronto Socorro de Porto Alegre Trauma and Burn Intensive Care Unit Rio Grande do Sul RS Brazil Trauma and Burn Intensive Care Unit, Hospital de Pronto Socorro de Porto Alegre - Rio Grande do Sul (RS), Brazil.; 3 Hospital de Terapia Intensiva Intensive Care Unit Teresina PI Brazil Intensive Care Unit, Hospital de Terapia Intensiva - Teresina (PI), Brazil.; 4 Instituto D’Or de Pesquisa e Ensino Rio de Janeiro RJ Brazil Instituto D’Or de Pesquisa e Ensino - Rio de Janeiro (RJ), Brazil.; 5 Hospital São José Unidade Local de Saúde de São José Lisboa Portugal Hospital São José, Unidade Local de Saúde de São José - Lisboa, Portugal.; 6 Nova Medical School Faculdade de Ciências Médicas de Lisboa Lisboa Portugal Faculdade de Ciências Médicas de Lisboa, Nova Medical School - Lisboa, Portugal.

In low- and middle-income countries, ensuring an adequate supply of critical care to meet increasing demand remains a challenge. In the last decades, substantial investment has increased bed availability in low- and middle-income countries. This pattern was even more pronounced in upper-middle-income countries such as Brazil.^(1)^ In this setting, the main problems are related to inequities driven by access, location, and payor rather than by absolute numbers representing supply itself. Upper-middle-income countries such as Brazil are not homogeneous; rather, they contain realities that mimic low-, middle-, and high-income settings.

By the end of 2025, Brazil had a total of 47,644 adult intensive care unit (ICU) beds, including 23,218 publicly funded beds within the Unified Health System (SUS - *Sistema Único de Saúde*) and 24,426 in the private sector. Between 2016 and 2025, the country increased its adult ICU bed capacity by 67.5%, rising from 28,441 to 47,644 beds. However, as illustrated in figure 1, this substantial expansion has not translated into equitable access, and marked disparities across sectors and regions continue to persist.

**Figure 1:**
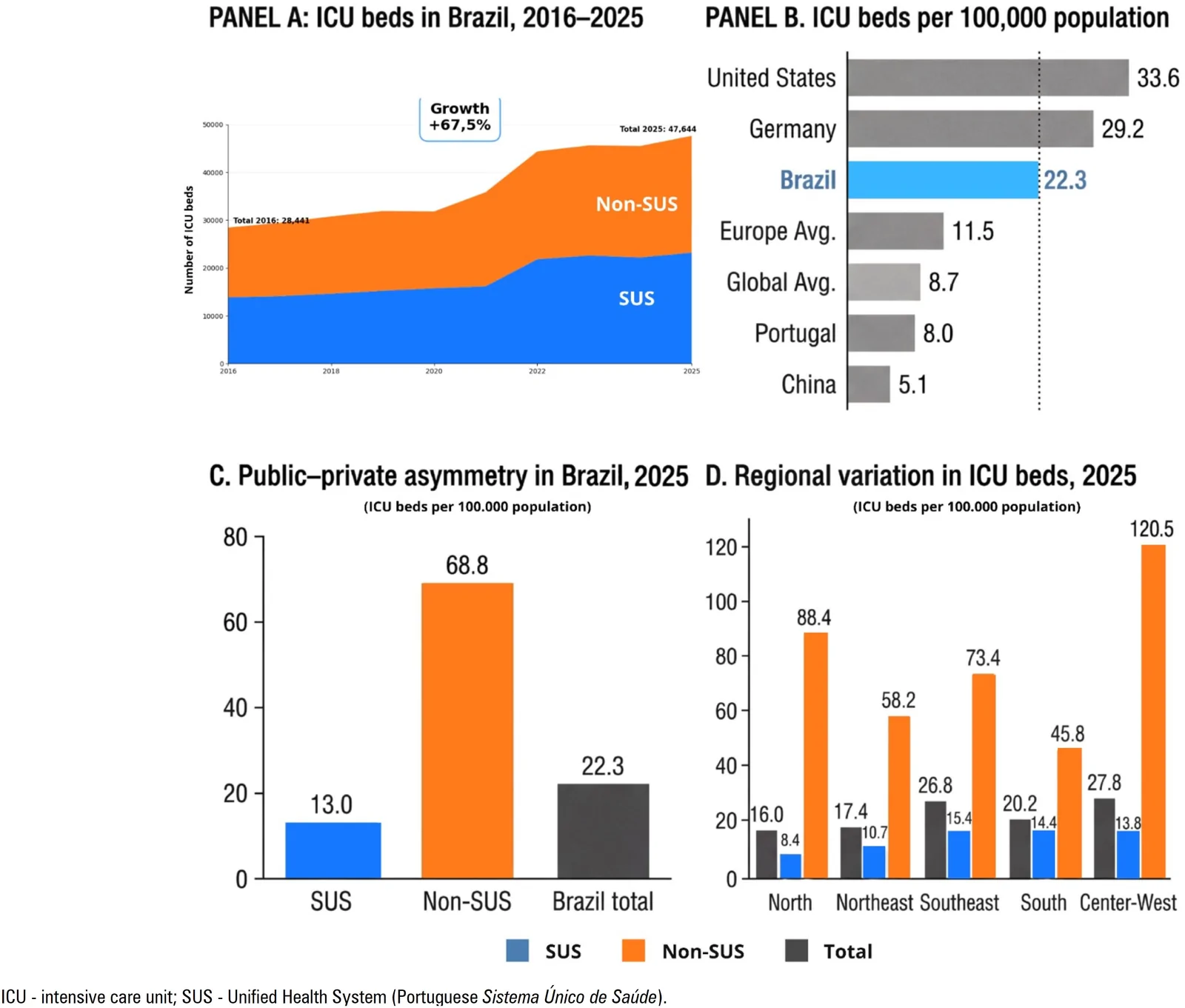
Beyond bed counts: intensive care unit capacity and access in Brazil.

(A) Availability in Brazil from 2016 to 2025, according to funding sources (Unified Health System *versus* non- Unified Health System); (B) Brazil in the international context: selected comparisons of adult intensive care unit bed capacity per 100,000 population; (C) public-private asymmetry in adult intensive care unit bed availability in Brazil in 2025; (D) regional variation in adult intensive care unit bed availability across Brazilian regions in 2025, overall and according to funding source.

Data sources: *Cadastro Nacional de Estabelecimentos de Saúde* (CNES - https://cnes.datasus.gov.br/pages/estabelecimentos/consulta.jsp); adult intensive care unit type I, II, and III; coronary intensive care unit type II and III), *Agência Nacional de Saúde Suplementar* (ANS - https://www.gov.br/ans/pt-br), and *Instituto Brasileiro de Geografia e Estatística* (IBGE - https://www.ibge.gov.br/). International estimates were derived from published reports and may reflect differences in intensive care unit definitions and reference years.

In terms of population density, this equates to 22.3 adult ICU beds per 100,000 population, 13.0 per 100,000 population for SUS users, and 68.8 per 100,000 population for private health insurance subscribers. In comparison to international norms, Brazil is well above the international average.^(2)^ Worldwide, the supply of ICU beds varies widely. A recent review found a mean of 8.7 ICU beds per 100,000 population worldwide.^(2)^ In contrast, the average supply of ICU beds in Europe is 11.5 beds per 100,000 population.^(3)^ In the United States, recent estimates range from 20 to 34 ICU beds per 100,000 population.^(4,5)^ In Asia, critical care supply is substantially lower at a median of 4.6 ICU beds per 100,000 population.^(6)^ In China, one of the world's largest absolute ICU bed supply figures, critical care supply has traditionally been low at 3.6 ICU beds per 100,000 population.^(6)^ Recent national data from China indicate that critical care supply is still on a growth trajectory, reaching 5.3 per 100.000 population in 2022.^(7)^ In Portugal, recent data show that critical care supply is at 8.0 active ICU beds per 100,000 population.^(8)^

Therefore, overall, Brazil ranks among the world's top countries in the number of adult ICU beds, behind only the United States and China. Yet this is far from a solution as it does not solve a fundamental weakness in how this capacity is distributed. As three out of every four Brazilians depend exclusively on public healthcare and have access to fewer than half of all available ICU beds, those in the private sector enjoy a bed density that is not only higher than the Brazilian average but also exceptionally high by international standards. Yet, in addition to this payor-based inequity, geographic location is a major issue in a large, unequally funded health system. In 2025, adult ICU bed density varied from 16.0 beds/100,000 in the North and 17.4 beds/100,000 in the Northeast to 26.8 beds/100,000 in the Southeast, 20.2 beds/100,000 in the South, and 27.8 beds/100,000 in the Center-West. As for the public sector (SUS network), access to ICU beds varied from 8.4 beds/100,000 in the North, and 10.7 beds/100,000 in the Northeast, to 15.4 beds/100,000 in the Southeast, and 14.4 beds/100,000 in the South, whereas 88.4 beds/100,000 in the North, and 120.5 beds/100,000 in the Center-West, were served within the private sector.

Overall, recent changes indicate that Brazil has significantly increased ICU bed availability but has not reduced its inequity.^(9)^

The distinction is one of clinical relevance, since ICU beds are not merely facilities but opportunities to obtain early, timely access to appropriate care for life-threatening conditions. A lower level of ICU accessibility is associated with worse outcomes.^(10)^ Likewise, delayed access to ICU beds has consistently been linked to adverse outcomes.^(11)^ On the other hand, a surplus of ICU beds is a challenge for staffing and optimal process of care adoption.^(12)^ As it can lead to low adherence to best practices and potentially an overutilization and increased healthcare costs, and admit those who are at low risk and derive no benefit.^(13,14)^

The next step in the development of policy in upper-middle-income countries such as Brazil in the realm of critical care should therefore go beyond bed counts. In the short term, the most significant advances in the public sector will likely come from better organization of available resources rather than the addition of new ones.^(12)^ In addition, the development of intermediate care facilities, improvements in governance, and adequate staffing should also be considered.^(12,15)^ Critical care in Latin America has shown that outcomes depend not only on the resources available but also on the organization and quality of care processes.^(11)^

The experience of Brazil is applicable to both upper-middle-income and high-income countries, with substantial lessons from the economic and health care perspectives. While the number of ICU beds remains a significant issue, equitable access to well-staffed ICU beds where evidence-based care is delivered in a timely manner should be the future of critical care policy development.

## Data Availability

The contents will be made available at the time of publication of the article.
